# Actions to respond to a troubled immigration environment

**DOI:** 10.3389/fpubh.2025.1517238

**Published:** 2025-09-19

**Authors:** Carol Galletly, Dulce Ruelas

**Affiliations:** ^1^Department of Psychiatry and Behavioral Medicine, Medical College of Wisconsin, Milwaukee, WI, United States; ^2^College of Nursing and Health Care Professions, Grand Canyon University, Phoenix, AZ, United States

**Keywords:** immigration policy, migrant health, migrant healthcare utilization, immigration environment, legislation

## Abstract

Migrants make up over 14% of the US population. Migrants’ perceptions and misperceptions of laws and impressions of the immigration environment influence their health and healthcare use. There are action steps that healthcare and public health providers can take to prevent migrants from avoiding care and to support their health in a troubled immigration environment. These include: making it clear that healthcare and public health entities do not have a role in immigration enforcement; communicating record transfer, access, and storage policies; clarifying rules regarding immigration penalties and health service use; and ensuring that migrants enjoy the right of confidentiality afforded to US residents. Public health representatives should expand preparedness efforts to include the potential for future hostile immigration-related discourse and legislation.

## Introduction

In 2023, an estimated 47.8 million foreign-born persons were living in the US making up 14.3 of the total population (1). The health of foreign-born persons living in the US is paramount, if only because they represent a substantial proportion of the population. Yet there is ample evidence that immigration laws and policies negatively impact the health of US migrants and deter their healthcare utilization (2–7).

### US immigration environment

Currently, immigration policy is a high priority among US lawmakers. Immigration figured largely in the Fall 2024 US presidential debates. During the debates, immigration was brought up 32 distinct times including in response to the moderator’s direct questions about the issue, amounting to an average of once every 6.5 min across the debates (8). On the first day of the current presidential administration, ten Executive Orders and Proclamations addressing immigration were issued (9, 10), and so far in the US 119th Congress (2025–2027), 250 immigration-related bills have been introduced (11).

Immigration is also a highly contentious issue in the US. A federal effort under the previous presidential administration to make substantial changes in immigration enforcement and support (12) often described as a bipartisan effort due to the dual-party nature of those who negotiated the legislation, led to even single-party coalitions fragmenting. Further, two of the three senators involved in negotiating the bill ultimately voted not to advance it (13). More recently, several Executive Orders have been challenged in US courts, one (14) with as many as 22 states blocking the executive action (15).

Despite considerable resistance to recent immigration enforcement actions (16), the current US immigration policy environment is decidedly hostile. Since January 20, 2025, there have been highly publicized immigration raids (17–19), and the detention of record or near-record numbers of migrants (20, 21), some of whom were lawfully present in the US (21). Indeed, in the first 100 days of the current US administration, over 66,000 migrants were detained and over 65,000 were removed from the US (22). Further, large groups of migrants have found their legal status challenged (23), and the militarization of immigration enforcement has been expanded (24–26).

### The health effects of a negative immigration policy environment

Unfortunately, political discourse addressing US immigration often focuses on the characterization of migrants themselves rather than on the arguably troubled US immigration system. In the Fall US presidential debates (8) and the subsequent Executive Orders promulgated by the 2025 US presidential administration (27–29), migrants were characterized as undesirable US residents, often persons who were criminals, gang members, and foreign adversaries.

This narrative of migrants as criminals and the subsequent efforts to deter migrants from coming to or remaining in the US was codified in the title of Arizona’s S. B. 1,070 “Support Our Law Enforcement and Safe Neighborhoods Act” (2010) (30). The rationale for the law, described as the “attrition through enforcement doctrine,” made explicit the intention to create an unwelcoming immigration environment. A controversial part of the law, the “show me your papers” provision requiring law enforcement officers to request federal registration papers from persons stopped or arrested who were suspected to be undocumented, was upheld by the US Supreme Court (31). Using 2009–2011 Behavioral Risk Factor Surveillance System (BRFSS) data, researchers conducted a natural experiment examining self-reported health before and after the provision became law. Researchers found that self-reported health status among Spanish speaking Arizona residents was poorer after the law was enacted and that self-reported health status was poorer between Arizona residents and residents of US-Mexico border states that had not enacted restrictive legislation (32). The mechanism for these negative effects on health is commonly attributed to stress (3, 33, 34).

In the Fall presidential debates, migrants were also characterized as burdens on US services, including health services (8). The narrative of migrants as burdens served as the foundation for a high-profile amendment to the national public charge rule (35) that greatly extended benefits for which a subset of migrants could be made ineligible for immigration advancement. The amended rule came into effect in the Fall of 2020 and was subsequently vacated in the Spring of 2021 (36). Researchers and health care providers noted declines in migrants’ utilization of public programs and services, including health programs and health services, even among those still eligible for services (37–39). For example, providers observed that migrants in Texas were opting not to reapply for Medicaid because of concerns over immigration consequences (37). Providers also noted that a subset of migrant parents had stopped taking their ill citizen children to public agencies or hospitals for healthcare due to fear that their personally identifying information would be shared with immigration officials (40). Importantly, many of these deleterious consequences emerged when the rule was circulated in draft form, well before the rule was even finalized and among persons who would be unaffected by it (41). Similarly, researchers noted a continued chilling effect on service use even after the restrictive rule was vacated (42, 43). Perceptions of immigration related laws and policies can influence behavior as much if not more than law “on the books,” especially in as dynamic an immigration environment as exists in the US (44).

### Expanded influence

An important consideration is that while most discussions of US immigration policy focus on undocumented migrants, those who are lawfully present and even those who are citizens are also affected. Many migrants live in mixed status households and fear deportation of loved ones (34, 45). Migrants with temporary legal status such as Temporary Protected Status (TPS) or with liminal immigration status such as Deferred Action for Childhood Arrivals (DACA) recipients may experience considerable stress as they grapple with changing immigration laws and fear losing their immigration status (46, 47). US citizens of non-European descent may be concerned that they will be caught up in the web of immigration law through racial profiling (48, 49). The net of persons who are affected by immigration laws and policies extends widely.

### Action steps

Our studies of the influence of actual and perceived immigration-related laws on Latino/a/é migrants’ health and utilization of health services highlight three areas of shared concern among Latino/a/é migrants that are pertinent here: concerns about collaboration between health service providers and immigration enforcement authorities (50–53); concerns about the confidentiality of personal information provided during healthcare and public health encounters (50–53); and concerns about eligibility for publicly-funded health services or the possibility of facing immigration penalties for using them (51, 54, 55). Although limited to Latino/a/é migrants, our findings may be instructive in the current immigration context and can inform steps for action.

We have found that among some groups of Latino/a/é migrants, there is the assumption that medical and public health personnel are required to report, or take the initiative to report, undocumented migrants to immigration authorities including Immigration and Customs Enforcement or ICE (50–53). Assumptions about collaboration between public health and healthcare entities and immigration officials are exacerbated by the fact that most public health agencies and many clinics that provide affordable care to migrants are funded by the government or are indeed government agencies (50). Public health representatives and healthcare providers must make it clear that public health and healthcare entities are not involved in immigration enforcement. Materials outlining clients’ rights should be disseminated in easily consumable documents in appropriate languages. It may also be helpful to avoid the appearance of government association, such as by including official insignia on forms and documents.

Concerns about the confidentiality of personally identifiable data emerged from findings in several of our studies (44, 51–53). Some groups of participants even believed that information given to healthcare or public health providers is routinely shared with immigration authorities (51, 52). Migrants should be assured that their personal information is not transferred from health departments or clinics to immigration authorities without their express permission, such as might be the case for transfer of vaccination records to civil surgeons. This, of course, is to the extent of current law and practice. Describing measures being done to protect the personal information of all clients may be helpful, and these are extensive in medical and public health settings. When possible, collecting information that may not be immediately relevant, especially about immigration status, should be avoided.

Still, unfortunately, recent data releases such as the Summer 2025 release of Medicaid data in several states, done expressly to identify undocumented migrants (56), suggest that migrant’s confidentiality concerns are founded. Providers should work to educate the public and law and policy makers about the chilling effect that data release may have on healthcare use among migrants. Providers should advocate for the enforcement of current laws and longstanding policies protecting the personal information of clients. They should also advocate for legislation that specifically addresses any weaknesses in current law.

Many participants shared concerns about being denied immigration advancement under the public charge rule if they utilize government services, including public insurance and health services (51–55). Some also assumed that they were ineligible for services to which they were entitled (50). Migrants need to be aware of the services for which they are eligible and that they may use without penalty. This will vary among migrants who reside in different US jurisdictions and who are in the US through different immigration channels (e.g., refugees, legal permanent residents, undocumented persons). Federally qualified health centers (FQHCs) are obligated to provide health services to all persons, regardless of immigration or insurance status, income, etc. These are located across the US, often in areas where need is concentrated, and can serve as a basic resource.

Laws, official policies, and practices related to confidentiality, eligibility for services, and permissible immigration enforcement actions are dynamic and vary among jurisdictions. To ensure that accurate information is being transferred to migrant clients, medical and public health providers should stay abreast of current laws, regulations, rulings, and actions. Providers may want to consult with attorneys specializing in immigration law and its administration. This can be costly. Agencies and clinics that are otherwise siloed should form coalitions to access this information jointly.

## Conclusion

It is likely that in the current contentious and dynamic immigration environment, negative characterizations of migrants and restrictive laws and policies will continue if not escalate. Public health personnel should expand preparedness efforts to address instances of heightened federal and state discourse and laws and policies that are unfavorable to migrants.
